# Changes in Child and Adult Care Food Program (CACFP) Practices at Participating Childcare and Education Centers in the United States Following Updated National Standards, 2017–2019

**DOI:** 10.3390/nu12092818

**Published:** 2020-09-15

**Authors:** Jamie F. Chriqui, Julien Leider, Rebecca M. Schermbeck, Anmol Sanghera, Oksana Pugach

**Affiliations:** 1 Division of Health Policy and Administration, School of Public Health, University of Illinois Chicago, Chicago, IL 60608, USA; 2 Institute for Health Research and Policy, University of Illinois Chicago, Chicago, IL 60608, USA; jleide2@uic.edu (J.L.); rscherm@uic.edu (R.M.S.); sangher2@uic.edu (A.S.); opugach@uic.edu (O.P.)

**Keywords:** CACFP, early childhood nutrition, meal patterns, nutrition policy

## Abstract

The U.S. Department of Agriculture’s (USDA) Child and Adult Care Food Program (CACFP) updated meal pattern standards took effect in October 2017. The aim of this quasi-experimental, pre-post study is to identify changes in food and beverage practices of CACFP-participating centers due to implementation of updated CACFP meal patterns over a 21-month period. Eight hundred and fifty-eight centers located in 47 states and the District of Columbia completed a survey (primarily electronic) at both time points (67.6% follow-up response rate). Multivariable logistic regressions with robust standard errors assessed changes over time, accounting for repeated observations within each site. From baseline to follow-up, centers reported the increased familiarity and implementation, albeit with time, money, and staffing-related challenges. Significant improvements were seen in not serving sugary cereals or flavored milk, in serving 100% whole grains, and serving processed meats less than once a week. While CACFP-participating centers reported making significant progress in meeting the updated meal pattern standards and suggested best practices within 15–19 months of their effective date, reported compliance and adherence to the standards and best practices was not universal. USDA, state agencies, and technical assistance providers should work to provide centers with additional guidance to help them with implementation.

## 1. Introduction

The Child and Adult Care Food Program (CACFP) is predominantly a program for low-income children and adults in the United States; approximately 81 percent of all meals served in CACFP in fiscal year 2019 were free or reduced-price meals [1]. For many children and adults receiving meals through CACFP, the program may provide the only nutritious meals that they receive on a given day. In 2016, the U.S. Department of Agriculture (USDA) updated the meal pattern standards governing CACFP. The updated standards took effect on 1 October 2017, although centers and homes participating in CACFP were provided a 1-year transition period for those entities making a “good faith effort” to comply with the standards [2,3]. In fiscal year 2019, nearly 4.8 million children and adults participated in CACFP [1]. Over 2 billion meals were served in CACFP homes, centers, and adult day care facilities during fiscal year 2019, with over 1.5 billion of these specifically being served in over 66 thousand CACFP-participating childcare centers [1,4]. Thus, the updates to the meal pattern standards have tremendous potential to reach millions of low-income persons.

The updated standards include requirements and best practices governing the foods and beverages served or offered to children while at a CACFP-participating location. The child and adult meal pattern requirements include: limiting the service of juice to once per day, requiring breakfast cereals to contain no more than six grams of sugar per dry ounce of cereal, limiting sugar in yogurt to no more than 23 g of sugar per six ounces of yogurt, allowing meat and meat alternatives to be served in place of the entire grain component at breakfast (up to three times per week), prohibiting flavored milk (2–5 years), requiring at least one serving of grain per day to be whole grain-rich, and requiring potable drinking water to be offered and available throughout the day, as well as other requirements. Additional best practices include: providing at least two servings of whole grain rich grains per day; limiting the service of processed meat to less than once per week; serving only natural, low fat cheese; making at least one of the two snack components a fruit or vegetable; providing at least one serving of dark green, red/orange, beans/peas (legumes), starchy, and other vegetables weekly, as well as other best practices. A reimbursable breakfast contains three components (fluid milk, vegetable/fruit, grains) and lunch and supper must contain five components (fluid milk, meat, vegetable, fruit, and grains) that meet the new meal pattern requirements [2].

The scientific literature focused on food and beverage practices amongst CACFP providers and/or implementation of CACFP standards/best practices has predominantly been within-state-specific. In Connecticut (CT), 256 non-CACFP and 87 CACFP-participating centers completed a survey during 2015–2016 (prior to the USDA updated standards) on the food environment for preschool children and adherence to CACFP nutrition standards. In CT, state licensing requires that all childcare providers implement CACFP meal pattern standards. CACFP-participating centers reported more nutritious food service than non-CACFP-participating centers, i.e., serving more fruit, whole grains, and low fat milk. Eighty-five percent of CACFP-participating centers were aware of the updates to the meal patterns [5]. A Georgia survey conducted in March–April 2017 (prior to the updated USDA standards) examined adherence to statewide beverage requirements among both CACFP and non-CACFP-participating childcare sites (centers and homes). They found better adherence to statewide beverage standards by CACFP-participating sites. Facilitators for implementing the requirement of limiting the service of juice to once a day for CACFP-participating sites included written guidelines on juice, family support for the guidelines, provider training, and information for the families [6].

Another state-specific study conducted in Nebraska from January through April 2017 (again, prior to the updated USDA standards) compares the nutrition-related best practices of 1170 CACFP and non-CACFP participating childcare centers and family childcare homes (FCCHs) in both rural and urban settings. In this study, the majority of providers (90%) reported increased implementation of nutrition best practices around serving fruits and vegetables at least one time per day and providing skim or fat-free milk to children. Centers in this study were less likely to implement practices that reduced or eliminated serving fried meats weekly. Reported barriers to implementing best practices include increased costs, time, and child preference for foods/beverages. Childcare centers (rural and urban) more often than FCCHs reported that lack of space to store food and lack of control over meals and snacks served as a barrier to implementing nutrition-related best practices [7].

Several studies conducted in 2016 examined food and beverage practices amongst CACFP vs. non-CACFP-participating providers in California (*n* = 680 overall). The first study found that compliance with most individual updated CACFP standards and best practices was high (>60%) but with all new standards was low (<23% of sites). Most survey respondents reported implementing the revised meal pattern standards as “not hard” overall. However, some barriers for specific standards were identified. For example, child and parent preferences were the most common barrier to limiting sugar in yogurt, while cost and being unsure of what to purchase were reported barriers to implementing the whole grain standard [8]. A second study, using the same sample, reported that CACFP sites were more likely than non-CACFP sites to provide nutritious foods and beverages [9]. Moreover, a third study assessed changes in beverages served in childcare sites following implementation of updated California beverage standards for licensed childcare providers. Overall, they found that adherence to the new standards improved over time (45.1% vs. 27.2%) and that CACFP sites were more adherent to the standards than non-CACFP sites (51.6% vs. 27.9%) [10].

Finally, a 2019 study in Delaware surveyed 137 CACFP sponsors one year after the implementation of the revised meal patterns. Eighty percent of these sponsors reported little to no challenges when implementing the changes to the CACFP program. Sponsors reported a high level of support for the changes by parents, and sponsors felt that there had been enough training to implement the changes. Sponsors also reported challenges to implementing the changes such as an increased cost to serve healthier foods and communicating with parents the changes to the program [11].

The current study builds upon the above literature as well as a baseline nationwide study that was conducted in late summer/early fall 2017 to assess readiness for early care and education (ECE) centers participating in CACFP to implement the updated standards. In the baseline study (*n* = 1343 respondents), the majority of centers reported being “very” familiar with the soon-to-be-implemented meal pattern updates. Most centers reported being “very” prepared to make the changes, had “very much” begun implementing the updates, and reported needing at least “some” additional time and funding to implement the standards. The majority of centers reported meeting many of the beverage standards/best practices, including fresh water availability, serving only unflavored milk, limiting juice, and never serving fruit drinks or regular soda. Most centers reported meeting the best practice for serving 100% whole grains (94.4%) and the majority of centers met the standards for sugars in cereal (69.9%). Roughly one third of centers reported serving only plain/unflavored yogurt or no yogurt, a conservative measure of meeting the yogurt standard. With regard to best practices, most centers reported serving snacks with at least one component being a fruit or vegetable (F/V) at least one time a day. Most centers also reported providing at least one serving of dark green, red/orange, starchy, and other vegetables at least once a week. Nearly 75% of centers reported serving processed meats less than once a week [12].

The purpose of the present study was to evaluate the short-term (15–19 months post the effective date) progress made in implementing the updated CACFP standards by ECE centers nationwide and to identify opportunities for continued progress going forward. We hypothesized that, from baseline to follow-up, as implementation of the updated standards took effect, there would be increases in centers’ familiarity with and reported implementation of the standards, that reported challenges would weaken over time, and that there would be improvements in the extent to which centers met the standards and best practices. To our knowledge, this is the first study to longitudinally examine changes in readiness and implementation of the updated CACFP standards by ECE centers across the United States. The study seeks to provide insight as to which aspects of the updated meal pattern standards have been easier for centers to implement as well as those aspects of the standards that may require further guidance from USDA and state agencies to facilitate implementation.

## 2. Materials and Methods

### 2.1. Study Design, Survey Administration, and Study Sample

This was a longitudinal, pre-post study of CACFP-participating ECE centers. A nationwide baseline survey of readiness for implementing the updated CACFP standards was conducted between August and September 2017. Detailed methods on the baseline survey are reported elsewhere [12]. Briefly, 1343 centers located in 47 states (including Alaska and Hawaii) and the District of Columbia (D.C.) responded to the baseline survey (25% response rate). In January 2019, a follow-up electronic survey was sent to 1341 of the baseline respondents (1 was determined closed as part of a separate sub-study and one was determined to only serve infants) via electronic mail. The survey was programmed and administered using a Research Electronic Data Capture (REDCap) database [13]. Both administrations of the survey (baseline and follow-up) were deemed exempt by the University of Illinois Chicago Institutional Review Board (protocol #2017-0549).

The 1341 baseline respondents were asked to complete a brief (15–20 min) follow-up survey and each respondent was offered the opportunity to receive a $20 electronic gift card following survey completion. An initial advance email was sent to the providers on 8 January 2019, letting them know that the survey would soon be sent out via electronic mail. The initial electronic survey was emailed on 15 January 2019 and follow-up reminders were sent weekly through 19 February 2019, and a final reminder was sent to partial responders on 4 March 2019. From 18 to 25 March 2019, a final mail-based survey was sent out to 545 non-respondents to the web-based survey with a postage-paid return envelope as well as a link to the web survey. (A copy of the mail survey is provided in the Supplementary Materials.) A final email reminder to non-responders to this mailing was sent on 17 April 2019. All centers were also offered the option of calling and completing the survey by phone. Through these attempts, 72 sites (5%) were deemed to have closed or merged or no longer participated in CACFP or only did so in summer (and therefore were ineligible). This left 1269 eligible centers. We used the American Association of Public Opinion Research’s (AAPOR) Outcome Rate Calculator for calculating the response rates [14]. In total, 858 centers (67.6%) located in 47 states (all except Arkansas, Louisiana, and Maine) and D.C. completed the follow-up survey; 788 of the responders (92%) used the web platform and an additional 70 responders (8%) completed the hard copy mail-back survey. Other centers either only partially completed the survey and their responses were not used (32 centers; 2.5%), declined (15 centers; 1.2%), logged on but did not complete any item (50 centers; 3.9%), or neither responded to the survey nor declined to take it (314 centers; 24.7%). The only statistically significant differences by response modality were for the updated standards requiring more staff (Internet: 54.83% vs. mail: 72.2%, *p* = 0.0175); not offering sugary cereals (Internet: 83.43% vs. mail: 60.81%, *p* < 0.001); meeting the dark green vegetable best practice (Internet: 60.9% vs. mail: 78.3%, *p* = 0.017), and meeting the starchy vegetable best practice (Internet: 66.84% vs. mail: 88.16%, *p* = 0.002).

At the outset, this study was designed to identify changes in food and beverage practices due to implementation of updated CACFP meal patterns. The study hypothesis was that there would be a 0.08 change in the types of foods/beverages served due to CACFP standards when comparing baseline (August–September 2017) to follow-up (January–May 2019) survey results. The sample size calculation to achieve 90% power with a two-sided hypothesis test at the 0.05 significance level was based on the test for two correlated proportions (McNemar test) for a pre-post design. Conservatively, we assumed 0.5 prevalence of each outcome at the baseline measurement and approximated the proportion of discordant pairs (the proportion of sites that changed response after intervention) as 0.5 using Machin and colleagues’ formula [15]. This indicated a required sample size of 840 sites in the longitudinal panel to detect a 0.08 difference in the outcome due to the intervention. Thus, with our 858 completed surveys at follow-up, we were able to exceed this threshold.

### 2.2. Survey Content

A copy of the follow-up survey is provided in the Supplementary Materials. The beginning section asked about the center name, zip, and confirming that the center still participated in CACFP as well as about the respondent role and how long they have been in their role. The next section asked about the center ownership, whether they had a food program sponsor, participated in Early Head Start or Head Start, and additional questions about their participation in CACFP including funding sources. The section also asked about meal and snack provision, preparation, and menus. The third section asked about training for the center and/or staff on CACFP and their staff’s perceived familiarity with and extent of implementing the revised meal pattern standards. The final section asked about specific meal and snack practices that were aligned with the CACFP standards and best practices. In total, the baseline survey included 101 questions and the follow-up survey included 116 questions, with 84 questions duplicative over the two time periods.

### 2.3. Measures

#### 2.3.1. Outcome Variable Measures

Three categories of outcome measures were obtained from the survey: implementation progress and challenges, meeting CACFP standards, and meeting CACFP best practices. Specific outcome measures for each category are briefly described herein. Specific question wording is provided in the survey included in the Supplementary Materials.

##### Implementation Progress and Challenges

Two items measured implementation progress: familiarity with the updated standards (follow-up survey question 26) and reported progress in implementing the standards (survey question 26b). Responses to both items were reported using a 4-item Likert-scale ranging from “very much” to “I don’t know.” For both items, the responses were dichotomized into 1 = “very much” and 0 = “somewhat,” “not at all,” or “I don’t know” responses. Four items measured perceived implementation challenges: amount of time needed to implement the standards (survey question 26c), implementation requires more work and resources than the site has with their current funding (question 26d), implementation requires more work and resources than the site has with current staffing (question 26e), and staff opposition to the revised standards (question 26f). Each of these questions was also measured using a 4-item Likert scale and then recoded into separate dichotomous measures as follows: time to implement (1 = “a lot of time” or “some time,” 0 = “not much time” or “I don’t know”), and additional funding needed, staffing needed, and staff opposition used the same dichotomization (1 = “very much” or “somewhat” and 0 = “not at all” or “I don’t know”).

##### Meeting CACFP Standards and Best Practices

Meeting the standards for making clean, fresh water available, never serving flavored milk, and serving 100% juice less than twice a day, and the best practice for never serving juice drinks and soda, was determined based on follow-up questions 27, 30, 31, and 32, respectively. Meeting the standards and best practices of only serving 1% or skim milk and no flavored milk was computed based on questions 29 and 30. Flavored milk never being served was identified based on question 30. Serving 100% juice less than twice a day was identified based on question 31. Whether sugar cereals were served was determined from questions 43a–f. Only serving plain/unflavored yogurt was identified based on question 41.

The best practice of the milk typically served being 1% or skim was identified based on follow-up question 29, as was whether whole milk was one of the milks typically served. Never serving fruit drinks was identified based on question 32. Best practice measures were computed for serving vegetables at least once a week separately for: dark green (based on spinach, question 39a1, and broccoli, question 39h1), red/orange (based on yams/sweet potatoes, question 39g1, and carrots, question 39i1), starchy (based on corn, question 39b1, mashed potatoes, question 39e1, potato wedges, question 39f1, and peas, question 39c1), and other vegetables (based on lettuce/packaged salads, question 39d1, cauliflower, question 39j1, mixed vegetables, question 39k1, and cucumbers, question 39l1). Serving a fruit or vegetable as a component of a snack at least once a day was identified based on question 40a. The best practice measure of serving 100% whole grains was identified based on questions regarding serving 100% whole wheat/whole grain bread (question 44a2), pasta (question 44b2), tortillas (question 44c2), and brown rice (question 44d). The CACFP best practice related to cheese encouraged only serving natural cheese or low fat/reduced fat cheese. The survey (question 42) only captured information on not serving cheese or serving ONLY low fat/reduced-fat cheese and, as such, we report on the question 42 measure herein. The best practice of serving processed meat less than once a week and whether any processed meat was served were identified based on questions regarding serving packaged lunch meat (follow-up survey question 45a1), beef or pork hot dogs (question 45b1), and turkey hot dogs (question 45c1).

#### 2.3.2. Control Variable Measures

Analyses controlled for a number of center characteristics. Control variables obtained from the survey included whether the center was corporate-owned, participated in Head Start/Early Head Start, was food program sponsored, the number of staff employed (categorized as 1–10, 11–20, 21–30, and ≥31 employees), enrolment capacity (categorized as 1–25, 26–50, 51–100, and 101–499 children), length of the center’s participation in CACFP (categorized as <10 and 10+ years), and the weekly rate for 2–5 year old children (categorized as free/no cost or state subsidized, $1–$100.99, $101–$200.99, and ≥$201). These items were only asked at baseline, except those relating to corporate ownership, Head Start/Early Head Start participation, and food program sponsorship, which were asked at both timepoints. Majority race (categorized as ≥50% non-Hispanic White, ≥50% non-Hispanic Black, ≥50% Hispanic, and mixed) and percent urban were obtained at the zip code level from the American Community Survey and 2010 Census data, respectively [16,17]. Data on whether state nutrition standards were linked to CACFP as of April 2016 were obtained from the Public Health Law Center [18]. Finally, census division was coded based on the state in which each center was located [19].

### 2.4. Statistical Analysis

Weights adjusting for non-response to both the baseline and follow-up surveys were developed. Weights developed for the baseline survey and described elsewhere [12] were multiplied by attrition weights designed to account for the probability of non-response to the follow-up survey to generate the final weights. The attrition weights were computed using a similar methodology. Specifically, the probability of attrition was computed for each center in the follow-up sample using logistic regressions including zip code-level characteristics and center characteristics from the baseline survey, using multiple imputation with chained equations with 200 imputations to impute missing center characteristics. Attrition weights were then computed based on the response rate within each decile of attrition probability. Final weights were trimmed at the 5th and 95th percentiles.

Using a generalized estimating equations (GEE) approach with an unstructured correlation structure, odds ratios and 95% confidence intervals were computed from multivariable logistic regressions with robust standard errors to assess changes over time accounting for repeated observations within site. Both unadjusted models, controlling only for time and division, and adjusted models that controlled for the center characteristics noted above, were estimated. Each model was restricted to a balanced sample of centers with data on the outcome and relevant control variables at both baseline and follow-up. Statistical significance was defined as a two-tailed *p*-value of less than 0.05. Analyses were conducted in Stata/SE 13.1. Specifically, *svy* commands were used to account for survey stratification and weighting in computing unadjusted prevalence estimates, while weights alone were included in GEE models, which are not supported with *svy* commands. Average adjusted prevalence estimates were computed from the GEE models using the *margins* command, which also took account of the weights [20].

## 3. Results

### 3.1. Characteristics of the Centers

Weighted characteristics of the centers participating at baseline and follow-up are detailed in Table 1. The majority of centers reported not being corporate-owned, Head Start/Early Head Start affiliated, or having a federal food program sponsor. Around three quarters of the centers employed 20 employees or less, while over three quarters of the centers reported having an enrolment capacity of 51 or more children. Over 63 percent of the centers had participated in CACFP for 10 years or more, and nearly one half of centers charged a weekly rate of between $101 and $200.99 for children aged 2–5 years old; nearly 21 percent of the centers did not charge or were state subsidized. Centers were located in zip codes that included predominantly White (>58%), predominantly Black (nearly 12%), predominantly Hispanic (>14%), or a mix of racial/ethnic identities amongst the residents. Centers were located in areas that were predominantly urban on average. Nearly 58 percent of the centers were located in a state with ECE standards that were linked to CACFP and the centers were located in all nine census divisions. Appendix A Table A1 provides unweighted characteristics of responders and non-responders for the follow-up survey along with corresponding *p*-values for whether the responders were statistically different from non-responders. As Appendix A Table A1 indicates, the responders and non-responders were not significantly different on any characteristic considered.

### 3.2. Implementation Progress and Challenges

**Figure 1:**
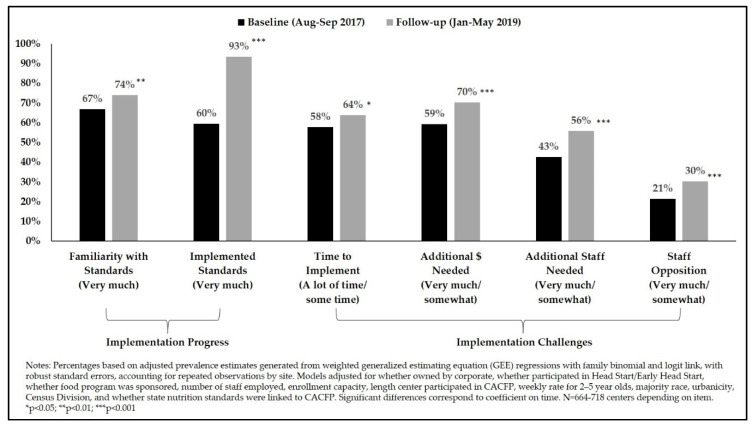
Progress and challenges in implementing updated CACFP meal pattern standards, 2017–2019.

At the time of the follow-up survey (conducted between 15 and 19 months following the effective date of the updated CACFP meal patterns), 74 percent of centers reported being very much familiar with the standards and 93 percent reported having implemented them (Figure 1). Centers were significantly more likely to report familiarity and implementation between the baseline and follow-up survey periods.

At the same time, the percentage of centers reporting challenges with needing more time, money, and staff to implement the updated meal patterns increased from baseline to follow-up (all *p*-values < 0.05). More than one half of the centers reported these challenges at follow-up (with 70 percent reporting needing more money to implement the standards as compared to only 59 percent at baseline). Reported staff opposition to the updated standards was lowest among the other reported challenges; however, significantly more centers did report staff opposition at follow-up as compared to the baseline period (21 percent at baseline vs. 30 percent at follow-up).

### 3.3. Progress in Meeting CACFP Standards

Table 2 presents the results of the adjusted regression models predicting compliance with selected standards, with statistically significant differences from baseline to follow-up noted with asterisks. (Appendix A Table A2 contains the unadjusted models.) At follow-up, most centers met the beverage overall standards/best practices (91.76%) and there was a significant increase in never serving flavored milk (baseline = 93.53%, follow-up = 96.32%). Although the percentage of centers reporting serving only 1% or skim milk and no flavored milk declined over time, this change was not significant. The percentage of centers that did not serve sugary cereals significantly increased by 12.8 percentage points (baseline = 67.56%, follow-up = 80.36%). Concomitant to the increase in not serving sugary cereals was a statistically significant decrease in centers reporting serving specific sugary cereals: Froot Loops (decline from 9.42% to 4.62%), Lucky Charms (decline from 5.63% to 2.79%), and Honey Nut Cheerios (decline from 23.83% to 14.42%).

### 3.4. Progress in Meeting Selected CACFP Best Practices

Results of the analyses examining centers’ progress in meeting CACFP best practices are presented in Table 3. (Appendix A Table A3 presents the unadjusted models.) There was a statistically significant decline in the prevalence of 1% or skim milk typically being served (from 86.19% to 80.76%) and a concomitant increase in the prevalence of whole milk being served (from 6.16% to 11.81%) from baseline to follow-up. There were no significant changes from baseline to follow-up in the serving of fruit drinks, any of the vegetable-related best practices, or including a fruit or vegetable as a component of a snack at least once a day. There was a significant increase in the prevalence of serving 100% whole grains over time (96.05% to 97.99%). There also were significant increases in not serving any cheese or only low fat/reduced fat cheeses and in meeting the best practice of serving processed meats (which tend to be high in sodium) less than one time per week. Notably, there also was a significant decline over time in centers serving any processed meats (91.79% to 68.49%).

## 4. Discussion

To our knowledge, this was the first pre-post nationwide panel study of implementation of the updated CACFP standards. The purpose of the present study was to evaluate the short-term progress made in implementing the updated CACFP standards by ECE centers nationwide and to identify opportunities for continued progress going forward. We hypothesized that, from baseline to follow-up, as implementation of the updated standards took effect, there would be increases in centers’ familiarity with and reported implementation of the standards, that challenges would weaken over time, and that there would be improvements in the extent to which centers met the standards and best practices. Our hypothesis that centers’ familiarity with and reported implementation of the standards would increase was proven accurate. By the time of follow-up, reported implementation was nearly universal (93% vs. 60% at baseline) and familiarity with the standards increased over time (from 67% to 74%).

However, and contrary to our hypothesis, we found that centers reported more challenges with needing more time, money, and staff to support implementation of the standards from baseline to follow-up. While these challenges are consistent with reported challenges that ECE centers face with CACFP generally [7,8,21], it is possible that, at the time of the baseline survey (which occurred immediately prior to the implementation effective date), centers did not yet have a sense of the resources that would be required for implementation. Once they began implementation, however, they may have realized that they needed more resources to support implementation. Another explanation may be associated with the timing for implementation. Although USDA granted a phased-in approach to CACFP providers that were making a “good faith” effort to begin implementation, ECE programs generally do not have the benefit of a standard “shutdown” period like schools do where they are typically closed during the school year and have time to train and implement new standards. Many childcare centers operate continuously throughout the year, without a clear stop and start date, which could make changes more difficult to implement. Finally, while school food service directors have reported similar challenges when implementing revisions to the National School Lunch Program standards [22,23], they have also noted that, over time, they have been able to minimize the barriers [23] and increase participation in the program [24].

Statistically significant improvements were seen in centers meeting some of the standards and best practices, specifically around flavored milk, cereals, and processed meats. It was particularly encouraging to see a significant decline in the proportion of centers reporting that they served flavored milk to children given that flavored milks are a significant source of sugary drinks among young children [25]. However, we also saw a concomitant decrease in the prevalence of the best practice of serving 1% or skim milk (decrease from 86.19% at baseline to 80.76% at follow-up) and a concomitant increase in the proportion of centers reporting that they served whole milk (increase from 6.16% to 11.81%). One possible explanation for the increase in whole milk being offered at ECE centers is at the request of parents and a reflection of a culture shift in dairy consumption in the United States. The 2019 annual milk sales data released by the USDA’s Agriculture Marketing Service reports an increase in full fat (whole) milk product sales despite a decrease in overall milk sales [26]. However, this finding warrants further exploration given that national nutrition recommendations and the CACFP meal pattern requirement are for children aged 2 to 5 years to be only served 1% or nonfat milk [2,25,27].

The food category with the lowest rate of reported compliance at baseline was the serving of sugary cereals [28]. At follow-up, the greatest improvements in compliance were with the reporting of not serving any sugary cereals (increase from 67.56% at baseline to 80.36% at follow-up). The reason for the dramatic increase in adherence to the requirement is unclear and is likely the result of many factors working together. Firstly, this was one of the easiest changes to make and likely took an initial period for centers to make the adjustment to their cereal procurement. Secondly, state agencies and sponsors likely conducted education for childcare providers during the transition period which contributed to the increased compliance [3,29]. Thirdly, throughout the U.S., there have been extensive public health campaigns to decrease the intake of added sugars and limit marketing of foods and beverages of poor nutritional quality, which include sugary cereals, to children [30,31]. More parents and ECE directors that understand and value the benefits of reduced added sugar in the diet makes policy changes easier to implement. As with beverages, sugary cereals are a significant contributor to young children’s consumption of added sugars [32,33,34]; thus, removing or reducing their availability in the ECE environment is key. Finally, the updated standards recommended that processed meats, which are typically high in sodium, be limited to less than one time per week. There was significant improvement in meeting this best practice from baseline to follow-up (72.66% to 78.48%) but, most notably, there was a significant reduction in the serving of any processed meats over time (from 91.79% to 68.49%). Given the sodium content of many processed meats, reducing their availability in ECE centers is another strategy for improving young children’s overall diet.

Overall, the finding of improvements in reported compliance with the updated standards is encouraging and similar to previously reported findings in the ECE space. In the only other study to assess longitudinal changes in food or beverage offerings among CACFP providers, Lee et al. assessed providers’ familiarity with and longitudinal implementation of a statewide beverage policy enacted in 2012 (that mirrored the USDA updated meal pattern standards) for all childcare centers in California [10]. Childcare centers in California reported an overall increased adherence to the four beverage provisions of the state’s law from 27.2% in 2012 to 45.1% in 2016 [10]. Given that our study was the first nationwide study, to our knowledge, to examine changes in compliance with the updated CACFP standards, it is encouraging that many centers are reporting compliance with the standards and best practices; future studies should continue to monitor compliance and examine the extent to which compliance continues to evolve over time.

### Limitations

The findings from this study should be considered within the context of the following limitations. Firstly, this was a quasi-experimental, pre-post study. Without a comparison or control group, such designs are typically considered to have numerous threats to internal and external validity. However, given that, from a federal perspective, the updated CACFP standards only apply to participating program providers and that the purpose of the study was to assess short-term changes in implementation of the updated federal CACFP standards, the use of a control or comparison group would not be appropriate. (We acknowledge that several U.S. states also require ECE providers to follow the CACFP standards regardless of CACFP participation status, but that was not the focus of this study.) Secondly, this was a study of CACFP-participating centers. Although centers serve the largest proportion of young children in ECE, FCCHs make up a larger proportion of CACFP providers (although the proportion has been declining in recent years) [4]. Ideally, future research will examine longitudinal implementation of the updated meal pattern standards in FCCHs. Thirdly, the study was based on survey self-report, which inherently is known to have numerous threats to internal validity but, given that the study included a nationwide sample of providers from 47 states and D.C. (including Alaska and Hawaii), obtaining objective measurements from a sample such as this would have been cost-prohibitive. Fourthly, although the baseline response rate was low (25%), it was comparable to the response rates for similar CACFP studies conducted within single states rather than nationally [8,35,36,37,38] and, more importantly, our response rate at follow-up was 67.6% of the baseline responders, which provided ample power to detect at least a 0.08 change for each outcome associated with the implementation of the standards. Finally, this study was conducted between 2017 and 2019, prior to the COVID-19 pandemic. Given that many ECE providers were shut down during the pandemic, the long-term impacts of these shutdowns on participation in CACFP and compliance with the standards will require concerted attention in the months and years to come.

## 5. Conclusions

In summary, there were improvements in meeting many of the revised CACFP meal pattern requirements and best practices during the first 15–19 months of their implementation. However, there continue to be opportunities to improve menus to adhere to the CACFP requirements and best practice recommendations more closely. Going forward, USDA and state agencies overseeing CACFP enrolment and compliance should focus on providing additional technical assistance and training to CACFP-participating centers to better position them to meet or exceed the standards and best practices so that all young children enrolled in these programs can benefit from the science-based, nutritionally balanced foods and beverages that the program is intended to provide.

## Figures and Tables

**Table 1:** Characteristics of the study sample.

Characteristic	% or Mean (SE)
Center characteristics	
Corporate-owned (follow-up; *n* = 858)	30.45 (1.83)
Participated in Head Start/Early Head Start (follow-up; *n* = 858)	33.03 (1.84)
Food program sponsored (follow-up; *n* = 856)	41.40 (1.94)
Number of staff employed (*n* = 853)	
1–10 employees	33.73 (1.84)
11–20 employees	38.98 (1.93)
21–30 employees	15.00 (1.36)
≥31 employees	12.29 (1.21)
Enrolment capacity (*n* = 841)	
1–25 children	7.00 (1.00)
26–50 children	17.45 (1.44)
51–100 children	40.28 (1.95)
101–499 children	35.27 (1.85)
Length of center’s participation in CACFP (*n* = 771)	
<10 years	36.92 (1.99)
10+ years	63.08 (1.99)
Weekly rate for 2–5-year-old children (*n* = 808)	
Free/no cost or state subsidized	20.86 (1.58)
$1–$100.99	12.83 (1.34)
$101–$200.99	49.16 (1.96)
≥$201	17.15 (1.33)
Zip code-level characteristics	
Majority (≥50%) race (*n* = 858)	
Non-Hispanic White	58.53 (1.96)
Non-Hispanic Black	11.55 (1.38)
Hispanic	14.12 (1.43)
Mixed	15.81 (1.47)
% Urban (Mean) (*n* = 858)	82.02 (1.07)
State/regional characteristics	
State nutrition standards linked to CACFP (*n* = 858)	
Not mentioned	42.25 (1.88)
Standards linked to CACFP	57.75 (1.88)
Census division (*n* = 858)	
New England	3.51 (0.17)
Middle Atlantic	11.79 (0.58)
East North Central	12.95 (0.49)
West North Central	6.82 (0.44)
South Atlantic	23.13 (0.61)
East South Central	6.76 (0.55)
West South Central	12.50 (0.65)
Mountain	5.46 (0.42)
Pacific	17.08 (0.64)

Notes: The overall sample included 858 centers located in 47 states and the District of Columbia. SE: standard error. CACFP: Child and Adult Care Food Program.

**Table 2:** Progress in meeting selected CACFP standards (adjusted models), 2017–2019.

Standard	Adjusted OR (95% CI)	Adjusted *p*-Value	Adjusted Prevalence (%)	
			Pre	Post
Beverage Standards				
Water availability, milk flavor, 100% juice all meet requirements, and site does not serve juice drinks or soda	1.41 (0.94–2.10)	0.094	88.98	91.76
Only 1% or skim milk served, and no flavored milk	0.85 (0.66–1.10)	0.222	80.54	78.10
Flavored milk is never served	1.88 * (1.03–3.41)	0.038	93.53	96.32
Sugar in Cereals Standard				
No sugar cereals served	2.08 *** (1.60–2.72)	<0.001	67.56	80.36
Frosted Flakes	0.74 (0.47–1.15)	0.184	8.32	6.38
Apple Jacks	0.58 (0.31–1.09)	0.091	6.17	3.76
Froot Loops	0.45 ** (0.28–0.73)	0.001	9.42	4.62
Sugar Smacks/Honey Smacks	NC	NC	NC	NC
Lucky Charms	0.47 ** (0.28–0.80)	0.005	5.63	2.79
Honey Nut Cheerios	0.51 *** (0.38–0.68)	<0.001	23.83	14.42

Notes: *n* = 689–714 centers. OR: odds ratio. CI: 95% confidence interval. NC: could not be computed (extremely high/low prevalence at both time points; see Appendix A for unadjusted model estimates). Odds ratios are from weighted generalized estimating equation (GEE) regressions with family binomial and logit link, with robust standard errors, accounting for repeated observations by site. Odds ratios correspond to coefficient on time. Models controlled for census division, whether owned by corporate, whether participated in Head Start/Early Head Start, whether food program was sponsored, number of staff employed, enrolment capacity, length of center’s participation in CACFP, weekly rate for 2–5-year-olds, majority race, urbanicity, and whether state nutrition standards were linked to CACFP. Average adjusted prevalence estimates were computed from the GEE models using the *margins* command in Stata. An additional model sought to examine changes in serving 100% juice less than twice a day but this model could not be computed (extremely high/low prevalence at both time points; see Appendix A for unadjusted model estimates). An additional model examined the extent to which centers were meeting the sugar in yogurt standard; the result was not significant and is not reported in the table for brevity. * *p* < 0.05; ** *p* < 0.01; *** *p* < 0.001.

**Table 3:** Progress in meeting selected CACFP best practices, 2017–2019.

Best Practice	Adjusted OR (95% CI)	Adjusted *p*-Value	Adjusted Prevalence (%)	
			Pre	Post
Beverage Best Practices				
Type of milk typically served is 1% or skim	0.66 ** (0.50–0.86)	0.002	86.19	80.76
Whole milk is one of milks typically served	2.09 *** (1.43–3.03)	<0.001	6.16	11.81
Site never serves fruit drinks	0.73 (0.40–1.32)	0.301	97.20	96.27
Other Best Practices				
Serves 100% whole grains	2.02 * (1.02–4.00)	0.043	96.05	97.99
Cheese not served, or only low fat/reduced fat cheese	1.35 * (1.07–1.69)	0.010	31.96	38.25
Processed meats served less than once a week	1.41 ** (1.10–1.80)	0.007	72.66	78.48
Any processed meats served	0.16 *** (0.12–0.23)	<0.001	91.79	68.49

Notes: *n* = 617–717 centers. OR: odds ratio. CI: 95% confidence interval. Odds ratios are from weighted generalized estimating equation (GEE) regressions with family binomial and logit link, with robust standard errors, accounting for repeated observations by site. Odds ratios correspond to coefficient on time. Models controlled for census division, whether owned by corporate, whether participated in Head Start/Early Head Start, whether food program was sponsored, number of staff employed, enrolment capacity, length of center’s participation in CACFP, weekly rate for 2–5-year-olds, majority race, urbanicity, and whether state nutrition standards were linked to CACFP. Average adjusted prevalence estimates were computed from the GEE models using the *margins* command in Stata. Additional models examined the extent to which centers were meeting selected vegetable and fruit best practices; none of the results were significant and, therefore, they are not reported in the table for brevity. * *p* < 0.05; ** *p* < 0.01; *** *p* < 0.001.

## Supplementary Materials

The following are available online at https://www.mdpi.com/2072-6643/12/9/2818/s1, Follow-up Survey Instrument.

## Appendix A. Supplementary Tables

**Table A1:** Unweighted descriptives, responders vs. non-responders.

Characteristic	Unweighted Responders	Unweighted Non-Responders	*p*-Value (Responders vs. Non-Responders)
Center characteristics			
Corporate-owned (baseline)	29.60	31.34	0.51
Participated in Head Start/Early Head Start (baseline)	33.57	34.43	0.75
Food program sponsored (baseline)	36.52	39.92	0.22
Number of staff employed			
1–10 employees	32.36	34.02	0.24
11–20 employees	37.63	41.08	
21–30 employees	16.65	14.32	
≥31 employees	13.36	10.58	
Enrolment capacity			
1–25 children	7.13	8.54	0.33
26–50 children	19.14	15.42	
51–100 children	39.24	40.63	
101–499 children	34.48	35.42	
Length of center’s participation in CACFP			
<10 years	35.28	40.10	0.10
10+ years	64.72	59.90	
Weekly rate for 2–5-year-old			
Free/no cost or state subsidized	22.03	21.80	0.64
$1–$100.99	12.38	12.36	
$101–$200.99	45.30	48.31	
≥$201	20.30	17.53	
Zip code-level characteristics			
% Urban (Mean)	80.24	80.98	0.66
Majority (≥50%) race			
Non-Hispanic White	68.30	63.71	0.28
Non-Hispanic Black	7.46	9.69	
Hispanic	11.07	12.99	
Mixed	13.17	13.61	
State/regional characteristics			
State nutrition standards linked to CACFP			
Not mentioned	40.33	42.06	0.53
Standards linked to CACFP	59.67	57.94	
Census division			
New England	5.59	3.09	0.39
Middle Atlantic	10.72	13.40	
East North Central	16.90	14.43	
West North Central	8.62	7.84	
South Atlantic	16.90	17.53	
East South Central	6.64	7.84	
West South Central	8.39	9.28	
Mountain	7.93	8.25	
Pacific	18.30	18.35	

Notes: *n* = 1343 centers in 47 states and D.C.; *n* = 1190–1343 due to missing data. Percentages or means shown. Percentages may not total 100% due to rounding. *p-*values are from Pearson’s chi-squared test for categorical variables or test of mean difference from Stata’s *lincom* command for continuous variables.

**Table A2:** Progress in meeting selected CACFP standards (unadjusted models), 2017–2019.

Standard	Unadjusted OR (95% CI)	Unadjusted *p*-Value	Unadjusted Prevalence, % (SE)	
			Pre	Post
Beverage Standards				
Water availability, milk flavor, 100% juice all meet requirements, and site does not serve juice drinks or soda	1.47 * (1.04–2.08)	0.031	88.90 (1.30)	92.12 (1.09)
Only 1% or skim milk served, and no flavored milk	0.83 (0.67–1.04)	0.102	80.23 (1.58)	77.16 (1.69)
Flavored milk is never served	1.83 * (1.11–3.01)	0.018	93.30 (1.01)	96.20 (0.76)
Serves 100% juice less than twice a day	NC	NC	97.03 (0.72)	99.36 (0.28)
Sugar in Cereals Standard				
No sugar cereals served	1.92 *** (1.52–2.43)	<0.001	70.14 (1.80)	81.50 (1.57)
Frosted Flakes	0.77 (0.52–1.14)	0.190	7.97 (1.05)	6.25 (0.94)
Apple Jacks	0.68 (0.41–1.16)	0.158	5.79 (0.94)	4.06 (0.82)
Froot Loops	0.48 *** (0.31–0.73)	<0.001	9.58 (1.19)	4.88 (0.88)
Sugar Smacks/Honey Smacks	NC	NC	0.41 (0.21)	1.80 (0.51)
Lucky Charms	0.58 * (0.36–0.94)	0.027	5.02 (0.84)	3.01 (0.67)
Honey Nut Cheerios	0.55 *** (0.43–0.71)	<0.001	21.49 (1.62)	13.28 (1.37)
Sugar in Yogurt				
Only plain/unflavored yogurt served	1.15 (0.98–1.35)	0.094	33.36 (1.91)	36.51 (1.95)

Notes: *n* = 821–851 centers. OR: odds ratio. NC: could not be computed (extremely high/low prevalence at both time points). Odds ratios are from weighted generalized estimating equation (GEE) regressions with family binomial and logit link, with robust standard errors, accounting for repeated observations by site. Odds ratios correspond to coefficient on time. Unadjusted models controlled for time and division. * *p* < 0.05; *** *p* < 0.001.

**Table A3:** Progress in meeting selected CACFP best practices (unadjusted models), 2017–2019.

Best Practice	Unadjusted OR (95% CI)	Unadjusted *p*-Value	Unadjusted Prevalence, % (SE)	
			Pre	Post
Beverage Best Practices				
Type of milk typically served is 1% or skim	0.67 *** (0.53–0.85)	<0.001	85.51 (1.40)	79.87 (1.62)
Whole milk is one of milks typically served	1.94 *** (1.38–2.72)	<0.001	6.53 (0.95)	11.87 (1.28)
Site never serves fruit drinks	0.83 (0.49–1.40)	0.487	97.28 (0.62)	96.75 (0.72)
Vegetable and Fruit Best Practices				
Dark green vegetables provided at least 1x/week	0.97 (0.81–1.15)	0.690	63.36 (1.89)	62.55 (1.94)
Red/orange vegetables provided at least 1x/week	0.84 (0.67–1.05)	0.126	77.33 (1.67)	74.19 (1.76)
Starchy vegetables provided at least 1x/week	1.00 (0.82–1.22)	0.992	69.12 (1.84)	69.09 (1.86)
Other vegetables provided at least 1x/week	0.80 * (0.64–1.00)	0.046	82.99 (1.46)	79.66 (1.61)
Fruit or vegetable is a component of a snack at least once a day, or every snack includes vegetable or fruit	1.09 (0.89–1.33)	0.393	60.96 (1.97)	62.94 (1.96)
Other Best Practices				
Serves 100% whole grains	2.07 * (1.05–4.06)	0.035	96.51 (0.72)	98.27 (0.54)
Only low fat/reduced fat cheese, or cheese not served	1.33 ** (1.09–1.61)	0.005	33.54 (2.01)	39.96 (2.11)
Processed meats served less than once a week	1.29 * (1.03–1.61)	0.026	74.68 (1.75)	79.08 (1.63)
Any processed meats served	0.19 *** (0.15–0.25)	<0.001	90.97 (1.09)	66.59 (1.87)

Notes: *n* = 713–854 centers. OR: odds ratio. Odds ratios are from weighted generalized estimating equation (GEE) regressions with family binomial and logit link, with robust standard errors, accounting for repeated observations by site. Odds ratios correspond to coefficient on time. Unadjusted models controlled for time and census division. * *p* < 0.05; ** *p* < 0.01; *** *p* < 0.001.
