# Supplementary material for: Changes in Child and Adult Care Food Program (CACFP) Practices at Participating Childcare and Education Centers in the United States Following Updated National Standards, 2017–2019
Source: Nutrients. 2020 Sep 15;12(9):2818. doi: 10.3390/nu12092818 (PMC7551123; doi:10.3390/nu12092818)
Supplement: Supplementary file 1 [file nutrients-12-02818-s001.pdf]

Site Id: (mail merge)

INSTITUTE FOR HEALTH RESEARCH AND POLICY
UIC

CCFP/FFP/CACFP Follow Up Survey

INSTRUCTIONS

Thank you for participating in this important study of child care sites. It may be helpful to consult with the person that is most familiar with the foods and beverages served at your child care site to complete Section C of this survey. Please mark only one answer for each question unless told otherwise and answer the questions straight through unless your selected response tells you to skip certain questions. As a reminder, your answers are completely confidential. We will never release your name, your child care site's name, or your sponsor's information to the public, to any state agency, or to our funder.

1. What is your name?

____

2. What is the name of your child care site? If you are a sponsor, please indicate the site for which you are completing the survey.

____

3. What zip code is your site located in?

____

4. What email address should we use for any future communications (including where the $20 Electronic Amazon gift card should be sent)?

____

5. Does your child care site still participate in the U.S. Department of Agriculture's Child and Adult Care Food Program (CACFP) also known as the Federal Food Program or Child Care Food Program (CCFP)?
- ☐ Yes
- ☐ No (**IF NO, SURVEY ENDS**)
- ☐ I don't know

6. What is your role at your current child care site? (Select all that apply.)
- ☐ Center Director/Assistant Director
- ☐ Center Owner/Franchisee
- ☐ Corporate/Sponsor
- ☐ CEO/COO/Administration *(e.g. Coordinators, Superintendent, Executive Director, Finance Directors, Program Supervisor, Office Manager)*
- ☐ CACFP Coordinator/Manager/Eligibility Director
- ☐ Food Service *(e.g. Manager, Cook, Coordinator)*
- ☐ Health and/or Nutrition Professional *(e.g. Child Nutrition Manager, Dietitian, Nutritionist, Health Specialist)*
- ☐ Teacher
- ☐ Other: (Please specify)

____

7. How long have you been in your role at your current child care site?
- ☐ <5 Years
- ☐ ≥5 Years - <10 Years
- ☐ ≥10 Years - <15 Years
- ☐ ≥15 Years
- ☐ I don't know

SECTION A. FEDERAL PROGRAM PARTICIPATION

Now we would like to learn more about your specific child care site.

8. Is your site corporate owned?
- ☐ Yes
- ☐ No
- ☐ I don't know

9. Is your site owned by a franchisee?
- ☐ Yes
- ☐ No
- ☐ I don't know

10. Does your site have a sponsor for the federal food program/CACFP/CCFP?
- ☐ Yes: (Please list)_______________________________________
- ☐ No
- ☐ I don't know

11. Does your site participate in the Early Head Start Program?
- ☐ Yes
- ☐ No
- ☐ I don't know

12. Does your site participate in the Head Start Program?
- ☐ Yes
- ☐ No
- ☐ I don't know

13. Does your site receive any additional funding? These are sources that are specific to the program not the child, such as grants from private donors, local organizations, or other state or federal money.
- ☐ Yes **(GO TO QUESTION 13a)**
- ☐ No
- ☐ I don't know

13a. Please select all additional program sources of funding that apply.
- ☐ Federal funding *(e.g. Child Care and Development Fund; I.D.E.A.; Military Child Development Program; Social Service Block Grant)*
- ☐ State Funding *(e.g. Race to the Top – Early Learning Challenge; Preschool Development Grant Program; State-Funded Pre-K Program; Title 1 Preschool)*
- ☐ Local Government Funding
- ☐ Community Organizations/Non-profits
- ☐ Private Donor/Funder
- ☐ Other: (Please list)

____

14. Who **PROVIDES meals**, such as breakfast and/or lunch for the children **ages 2-5** at your site?
- ☐ The site provides all meals
- ☐ Combination of site providing meals and children bring meals from home
- ☐ The children bring all meals from home **(GO TO QUESTION 16)**
- ☐ No meals are served or provided on site **(GO TO QUESTION 16)**

14a1. Who **PREPARES** the **meals** for the children **ages 2-5** at your child care site? (Select all that apply.)

- ☐ Site Staff **(GO TO QUESTION 14b1)**
- ☐ Central Kitchen/Sponsor delivers to the site
- ☐ Catering Company/Vendor
- ☐ Hospital/Nursing Home/Senior Dining Center
- ☐ School District
- ☐ Other: (Please list) ___________________________________

(GO TO QUESTION 15)

14b1. What is the training of the person preparing the **meals** at your site? (Select all that apply.)

- ☐ Food Sanitation License
- ☐ Registered Dietitian
- ☐ CACFP Training *(e.g. Annual conference, webinars)*
- ☐ In House Training *(e.g. training from someone on-site such as an administrator, staff dietitian, staff food program manager or another center staff, "on the job training")*
- ☐ Experience
- ☐ Food Manager Training Certificate/License
- ☐ Nutrition or Food Program/Safety Training Course/Certificate
- ☐ State/Federal Training
- ☐ None
- ☐ Other: (Please specify) _______________________________
- ☐ I don't know

15. Where are the foods and beverages for your **meals** purchased? (Select all that apply.)

- ☐ Food Service Provider *(e.g. Sysco, Sodexho, US Food Service)*
- ☐ Warehouse Store *(e.g. Sam's Club, Costco)* **(GO TO QUESTION 15a2)**
- ☐ Local Grocery Store **(GO TO QUESTION 15a2)**
- ☐ Amazon Pantry
- ☐ Other Online Grocery Store *(e.g. Peapod, Penny Juice)*
- ☐ Catering Company
- ☐ Food Bank/Pantry **(GO TO QUESTION 15a2)**
- ☐ Local Farmers *(e.g. Farmers market, CSA, local farms/farmers)* **(GO TO QUESTION 15a2)**
- ☐ School District **(GO TO QUESTION 15a2)**
- ☐ Other: (Please specify) ________________________________
- ☐ I don't know **(GO TO QUESTION 15a2)**

15a2. Approximately, how far in miles do you travel to purchase food and beverages for your site?

____

16. Family style meal service is a type of meal service that allows children to serve themselves from communal plates of food with assistance from supervising adults, if needed. Does your site use this type of meal service for children ages 2-5 years?

- ☐ Yes
- ☐ No
- ☐ I don't know

17. Who **PROVIDES** **snacks** for the children **ages 2-5** at your site?

- ☐ The site provides all snacks
- ☐ Combination of site providing snacks and children bring snacks from home
- ☐ The children bring all snacks from home **(GO TO QUESTION 18)**
- ☐ No snacks are served or provided on site **(GO TO QUESTION 18)**

17a1. Who **PREPARES** the **snacks** for the children **ages 2-5** at your child care site? (Select all that apply.)

- ☐ Site Staff
- ☐ Central Kitchen/Sponsor delivers to the site
- ☐ Catering Company/Vendor
- ☐ Hospital/Nursing Home/Senior Dining Center
- ☐ School District
- ☐ Other: (Please specify) ___________________________________

18. Who prepares the **menus** for your site? (Select all that apply.)

- ☐ Corporate Office **(GO TO QUESTION 18b)**
- ☐ Food Program/CACFP/CCFP Sponsor **(GO TO QUESTION 18b)**
- ☐ Meal Planning Service *(e.g. MinuteMenu, Procare Software)* **(GO TO QUESTION 18b)**
- ☐ Center Director/Assistant Director
- ☐ On-site Kitchen/Food Manager/Cook *(e.g. Chef, cook, food program specialist/manager)*
- ☐ Administration *(e.g. Owner/co-owner, Office Manager, Central Office Food Coordinator; Executive Director/not on site director)*
- ☐ Food Service Provider/Vendor
- ☐ Health and/or Nutrition Professional *(e.g. Dietitian, dietitian reviews menus, dietitian with another person/group, health services coordinator)*
- ☐ School District
- ☐ Other: (Please specify) ___________________________________
- ☐ I don't know

18b. Do you or are you allowed to make changes to the menus?

- ☐ Yes
- ☐ Only with Corporate, Sponsor, or Meal Planner Service Approval
- ☐ No
- ☐ I don't know

19. How often do you make substitutions/changes to the posted or planned menu?

- ☐ More than once a week
- ☐ Once a week
- ☐ 2-3 times a month
- ☐ Once a month or less
- ☐ Never
- ☐ I don't know

20. Do you use the USDA Food Buying Guide Mobile Application to help guide your food and beverage purchasing for your child care site? https://www.fns.usda.gov/tn/food-buying-guide-mobile-app

- ☐ Yes
- ☐ No
- ☐ I don't know

21. Do you use any WIC resources to decide what to purchase for your center?

- ☐ Yes: (Please list) ____
- ☐ No
- ☐ I don't know

SECTION B. FEDERAL FOOD PROGRAM/CACFP/CCFP-SPECIFIC QUESTIONS

Now we would like to ask a few questions about your site's participation in the Federal Food Program/CACFP/CCFP specifically. It may help to have the person who is responsible for the food program at YOUR SITE, help you to respond to these questions if it is someone other than yourself.

22. Does your site receive training on the federal food program/CACFP/CCFP?
    - ☐ Yes, annually
    - ☐ Yes, semi-annually
    - ☐ Yes, quarterly
    - ☐ Other
    - ☐ No **(GO TO QUESTION 23)**
    - ☐ I don't know **(GO TO QUESTION 23)**

22a1. Who provides you/your site with the training for the federal food program/CACFP/CCFP? (Select all that apply.)
- ☐ Food Program Sponsor
- ☐ State Agency
- ☐ Corporate
- ☐ Franchisee
- ☐ Menu Planning Service
- ☐ USDA's Food and Nutrition Service (FNS)
- ☐ USDA's Team Nutrition
- ☐ Institute of Child Nutrition (ICN)
- ☐ Head Start Program
- ☐ Health and/or Nutrition Professional *(e.g. Health Services Coordinator/Manager/Director, Agency Food Service and Nutrition staff, contracted dietitian/RD, health and nutrition consultant)*
- ☐ Site Level Training *(e.g. Director/manager, admin assistant/staff, child development manager, owner, fiscal/HR, office manager, program staff/director/manager, we train our staff, in house, onsite training)*
- ☐ CACFP
- ☐ Food Service/Vendor/Catering Company *(e.g. director of food services, food coordinator, food manager, food program manager, food service manager, food distributor, head cook; vendor; catering company)*
- ☐ Formal Conference/Webinar/Online Training *(e.g. E-learning website, ISBE webinars, various conference and websites)*
- ☐ Other: (Please specify) ______________________________
- ☐ I don't know

23. If you work with a menu planning program/service such as MinuteMenu or Procare Service, do they provide you with training on the federal food program/CACFP/CCFP?
    - ☐ Yes
    - ☐ No
    - ☐ I do not use a menu planning program/service
    - ☐ I don't know

24. Who do you contact if you have questions about the federal food program/CACFP/CCFP? (Select all that apply.)
    - ☐ Food Program Sponsor
    - ☐ State Agency
    - ☐ Corporate
    - ☐ Franchisee
    - ☐ Menu Planning Service
    - ☐ USDA's Food and Nutrition Service (FNS)
    - ☐ USDA's Team Nutrition
    - ☐ Institute of Child Nutrition (ICN)
    - ☐ Head Start Program
    - ☐ Health and/or Nutrition Professional *(e.g. Health Services Coordinator/Manager/Director, Agency Food Service and Nutrition staff, contracted dietitian/RD, health and nutrition consultant)*
    - ☐ Site Level Training *(e.g. Director/manager, admin assistant/staff, child development manager, owner, fiscal/HR, office manager, program staff/director/manager, we train our staff, in house, onsite training)*
    - ☐ CACFP
    - ☐ Food Service/Vendor/Catering Company *(e.g. director of food services, food coordinator, food manager, food program manager, food service manager, food distributor, head cook; vendor; catering company)*
    - ☐ Formal Conference/Webinar/Online Training *(e.g. E-learning website, ISBE webinars, various conference and websites)*
    - ☐ Other: (Please specify) ___________________________________
    - ☐ I don't know

25. Who does compliance checks for the federal food program/CACFP/CCFP for your site? (Select all that apply.)
    - ☐ Corporate Office
    - ☐ Food Program Sponsor
    - ☐ The State through an audit
    - ☐ CACFP *(not as part of a regular audit)*
    - ☐ Finance/Accounting
    - ☐ Food Service
    - ☐ Head Start
    - ☐ Health and/or Nutrition Professional
    - ☐ Self-Audit
    - ☐ Other: (Please specify) ___________________________________
    - ☐ I don't know

26. Are you familiar with the **revised** federal food program/CACFP/CCFP standards regarding foods and drinks served at childcare sites that took effect October 2017?
    - ☐ Very Much
    - ☐ Somewhat
    - ☐ Not at all **(GO TO SECTION C)**
    - ☐ I don't know **(GO TO SECTION C)**

26a. From whom has your site received **training on** the **revised** federal food program/CACFP/CCFP standards? (Select all that apply.)

- ☐ Food Program Sponsor
- ☐ State Agency
- ☐ Corporate
- ☐ Franchisee
- ☐ Menu Planning Service
- ☐ USDA's Food and Nutrition Service (FNS)
- ☐ USDA's Team Nutrition
- ☐ Institute of Child Nutrition (ICN)
- ☐ Site Level Training *(e.g. Director/manager, admin assistant/staff, child development manager, owner, fiscal/HR, office manager, program staff/director/manager, we train our staff, in house, onsite training)*
- ☐ CACFP
- ☐ Food Service/Vendor/Catering Company
- ☐ Formal Conference/Webinars/Online Training *(e.g. E-learning website, ISBE webinars, various conference and websites)*
- ☐ Head Start Program
- ☐ Health and Nutrition Professional *(e.g. Health Services Coordinator/Manager/Director, Agency Food Service and Nutrition staff, contracted dietitian/RD, health and nutrition consultant)*
- ☐ Other: (Please specify) ___________________________________
- ☐ I don't know
- ☐ I/we have not received training

26b. To what extent has your site implemented the **revised** federal food program/CACFP/CCFP standards in your childcare site?

- ☐ Very Much
- ☐ Somewhat
- ☐ Not at all
- ☐ I don't know

26c. How much **time** do you think it has taken you **to put** the **revised** federal food program/CACFP/CCFP standards in place at your child care site?

- ☐ A Lot of Time
- ☐ Some Time
- ☐ Not Much Time
- ☐ I don't know

26d. To what extent do you think the **revised** federal food program/CACFP/CCFP standards have required more work and resources than your site has with current **funding**?

- ☐ Very Much
- ☐ Somewhat
- ☐ Not at all
- ☐ I don't know

26e. To what extent do you think the **revised** federal food program/CACFP/CCFP standards have required more work and resources than your site has with current **staffing**?

- ☐ Very Much
- ☐ Somewhat
- ☐ Not at all
- ☐ I don't know

26f. To what extent do you think site **staff have been opposed** to implementing the **revised** federal food program/CACFP/CCFP standards?

- ☐ Very Much
- ☐ Somewhat
- ☐ Not at all
- ☐ I don't know

26g. To what extent has it been difficult to get some of the foods specified in the **revised** federal food program/CACFP/CCFP?

- ☐ Very Much
- ☐ Somewhat
- ☐ Not at all
- ☐ I don't know

SECTION C. ABOUT MEALS AND SNACKS

YOU ARE ALMOST DONE! Finally, we would like to learn more about the foods and beverages provided at your site. Please have the person preparing your meals either assist you in completing this section or have them complete this section for you. The following questions pertain to **children ages 2-5 years**.
*Section C should only be completed where the site provides meals and/or snacks (see Questions 14 and 16).

27. Is clean, fresh water available to children upon request throughout the day such as from a water fountain or in a cup?
   - ☐ Yes
   - ☐ No **(GO TO QUESTION 28)**
   - ☐ I don't know

27a. What is the source of the drinking water available to children at your child care site? (Select all that apply.)

- ☐ Tap Water
- ☐ Filtered Tap Water
- ☐ Bottled Water
- ☐ I don't know

28. Is the site's tap water tested for contaminants?
   - ☐ Yes
   - ☐ No
   - ☐ I don't know

29. What type of milk does your site TYPICALLY serve to children ages **2-5 years**? (Select all that apply.)
   - ☐ Whole Milk
   - ☐ Reduced-Fat (2%) Milk
   - ☐ Low-Fat (1%) Milk
   - ☐ Fat-Free (Skim) Milk
   - ☐ I don't know

30. How often does your site serve flavored milk to children ages **2-5 years**?
   - ☐ More than twice a day
   - ☐ Twice a day
   - ☐ Once a day
   - ☐ Less than once a day
   - ☐ Never
   - ☐ I don't know

31. How often does your site serve 100% juice as labeled on the package to children ages **2-5 years**?
   - ☐ More than twice a day
   - ☐ Twice a day
   - ☐ Once a day
   - ☐ Less than once a day
   - ☐ Never **(GO TO QUESTION 32)**
   - ☐ I don't know

31a. How do you know the juice is 100% juice? (Select all that apply.)

- ☐ By reading the ingredient list/label
- ☐ Food Program Sponsor
- ☐ Menu Planning Service
- ☐ On-site Kitchen/Food Manager/Cook
- ☐ I am not sure. I just think it is 100% juice.

32. How often does your site serve fruit drinks or fruit-flavored beverages other than 100% fruit juice such as Sunny Delight, Hi-C, Kool Aid, or lemonade to children ages **2-5 years**?
- ☐ All the time
- ☐ Sometimes
- ☐ Rarely
- ☐ Never
- ☐ I don't know

33. What beverage is TYPICALLY provided to children ages **2-5 years** enrolled at your site for **breakfast**? (*By typical, we mean the beverage that is provided most often or on a regular basis to children as the "default" beverage option. This does not include special requests or accommodations for allergies, etc.)*
- ☐ Water
- ☐ Unflavored Low-fat (1%) or non-fat (skim) milk
- ☐ Unflavored 2% (reduced fat) milk
- ☐ Unflavored Whole milk
- ☐ 100% juice
- ☐ Juice Drinks
- ☐ Other (Please list): ___________________________________

34. What b beverage is TYPICALLY provided to children ages **2-5 years** enrolled at your site for **lunch**? (*By typical, we mean the beverage that is provided most often or on a regular basis to children as the "default" beverage option. This does not include special requests or accommodations for allergies, etc.)*
- ☐ Water
- ☐ Unflavored Low-fat (1%) or non-fat (skim) milk
- ☐ Unflavored 2% (reduced fat) milk
- ☐ Unflavored Whole milk
- ☐ 100% juice
- ☐ Juice Drinks
- ☐ Other (Please list): ___________________________________

35. What beverage is TYPICALLY provided to children ages **2-5 years** enrolled at your site for **snacks**? (*By typical, we mean the beverage that is provided most often or on a regular basis to children as the "default" beverage option. This does not include special requests or accommodations for allergies, etc.)*
- ☐ Water
- ☐ Unflavored Low-fat (1%) or non-fat (skim) milk
- ☐ Unflavored 2% (reduced fat) milk
- ☐ Unflavored Whole milk
- ☐ 100% juice
- ☐ Juice Drinks
- ☐ Other (Please list): __________________________________

36. How often does your site serve fresh fruits such as cut apple, banana, or orange slices?
- ☐ More than twice a day
- ☐ Twice a day
- ☐ Once a day
- ☐ Less than once a day
- ☐ Never
- ☐ I don't know

37. How often does your site serve canned fruits in 100% juice to children ages **2-5 years** such as canned pineapple, canned peaches, or fruit salad?
- ☐ More than twice a day
- ☐ Twice a day
- ☐ Once a day
- ☐ Less than once a day
- ☐ Never **(GO TO QUESTION 38)**
- ☐ I don't know **(GO TO QUESTION 38)**

37a. How does your site determine the canned fruit is in 100% juice? (Select all that apply.)
- ☐ By reading the ingredient list/label
- ☐ Food Program Sponsor
- ☐ Menu Planning Service
- ☐ On-site Kitchen/Food Manager/Cook
- ☐ I am not sure. I just think it is in 100% juice.

38. How often does your site serve thawed frozen fruit to children ages **2-5 years** such as thawed mango, pineapple chunks, mixed fruit, or berries?
- ☐ More than twice a day
- ☐ Twice a day
- ☐ Once a day
- ☐ Less than once a day
- ☐ Never
- ☐ I don't know

PLEASE USE THE TABLE TO COMPLETE QUESTION 39

39. Does your site provide any of the following vegetables (fresh, frozen, or canned) to children ages **2-5 years**? (Select all that apply.)
    - ➢ For every vegetable selected in question 39, answer the question “How often does your site provide [the selected vegetable] to children ages 2-5 years?” with the answers of “more than once a week”, “once a week”, “less than once a week”, “I don’t know”.

39. Does your site provide…(select all that apply)		For each vegetable provided, how often is it provided?			
		More than once a week	Once a week	Less than once a week	I don’t know
☐	a. Spinach	☐	☐	☐	☐
☐	b. Corn	☐	☐	☐	☐
☐	c. Peas	☐	☐	☐	☐
☐	d. Lettuce/packaged salad	☐	☐	☐	☐
☐	e. Mashed potatoes	☐	☐	☐	☐
☐	f. Potato wedges	☐	☐	☐	☐
☐	g. Yams/Sweet potatoes	☐	☐	☐	☐
☐	h. Broccoli	☐	☐	☐	☐
☐	i. Carrots	☐	☐	☐	☐
☐	j. Cauliflower	☐	☐	☐	☐
☐	k. Mixed vegetables	☐	☐	☐	☐
☐	l. Cucumbers	☐	☐	☐	☐
☐	m. Beans/legumes (i.e. pinto beans, black beans, refried beans, lentils, hummus, edamame)	☐	☐	☐	☐
☐	n. Other (Fill in blank):				
☐	o. We do not serve vegetables.				

40. How many snacks are offered per day at your site?
    - ☐ More than two
    - ☐ Two
    - ☐ One
    - ☐ None **(GO TO QUESTION 41)**
    - ☐ I don’t know

40a. How often is a fruit or vegetable one of the components of a **snack** offered at your site to children ages **2-5 years**?
- ☐ Every snack has either a vegetable or fruit as part of it
- ☐ Once a day
- ☐ Once a week
- ☐ Less than once a week
- ☐ Never
- ☐ I don’t know

41. What type of yogurt does your site serve to children ages **2-5 years**? (Select all that apply.)
    - ☐ Fruit flavored yogurt (e.g. blueberry, strawberry)
    - ☐ Vanilla yogurt
    - ☐ Plain/Unflavored yogurt
    - ☐ Other flavored yogurt: (Please list) ___________________________
    - ☐ We don’t serve yogurt **(GO TO QUESTION 42)**
    - ☐ I don’t know **(GO TO QUESTION 42)**

41b. What is the brand and type of yogurt most often served to children ages **2-5 years** at your site? If you do not know, please write “I don’t know”.
____

42. When your site serves cheese to children ages **2-5 years**, does your site serve low fat or reduced fat cheese?
    - ☐ All the time
    - ☐ Sometimes
    - ☐ Rarely
    - ☐ Never
    - ☐ We do not serve cheese
    - ☐ I don’t know

PLEASE USE THE TABLE TO COMPLETE QUESTION 43

43. Does your site provide the following cereals (name brand or store brand) to children **ages 2-5 years**? (Select all that apply.)
    - ➢ For every cereal selected in question 43, answer the question “How often does your site provide [the selected cereal] to children ages 2-5 years?” with the answers of “more than once a week”, “once a week”, “less than once a week”, “I don’t know”.

43. Does your site provide…(select all that apply)		For each cereal provided, how often is it provided?			
		More than once a week	Once a week	Less than once a week	I don’t know
☐	a. Frosted Flakes	☐	☐	☐	☐
☐	b. Apple Jacks	☐	☐	☐	☐
☐	c. Froot Loops	☐	☐	☐	☐
☐	d. Sugar Smacks/Honey Smacks	☐	☐	☐	☐
☐	e. Lucky Charms	☐	☐	☐	☐
☐	f. Honey Nut Cheerios	☐	☐	☐	☐
☐	g. Multigrain Cheerios	☐	☐	☐	☐
☐	h. Cheerios (original)	☐	☐	☐	☐
☐	i. Rice Krispies	☐	☐	☐	☐
☐	j. Kix	☐	☐	☐	☐
☐	k. Shredded Wheat (non-frosted)	☐	☐	☐	☐
☐	l. Frosted Shredded Wheat	☐	☐	☐	☐
☐	m. Other (Fill in blank):				
☐	n. We do not serve cereal.				

PLEASE USE THE TABLE TO COMPLETE QUESTION 44

44. Which products does your site provide **children ages 2-5 years**? (Select all that apply.)
    - 44.1 For each grain product selected in question 44, answer the question "How often does your site provide [the selected grain product] to children ages 2-5 years?" with the answers "Twice a day or more", "Once a day", "2-3 times per week", "Once a week", "Less than once a week", or "I don't know".
    - 44.2 For whole wheat bread, whole wheat pasta, and whole wheat tortilla selected in question 44, answer the question "Is the [selected grain product product] 100% whole wheat or whole grain?" with the answers "Yes", "No", or "I don't know".
    - 44.3 For a "Yes" response in question 44.2, please answer the question "How do you know the [selected grain product] is 100% whole wheat or whole grain?" (Select all that apply.) with the answers "By reading the ingredient list/label", "Food Program Sponsor", "Menu Planning Service", "On-site Kitchen/Food Manager/Cook", "I am not sure. I just think it is 100% whole wheat/grain.", and/or "The product is darker in color." Then move on to the next grain product in the list.
    - 44.4 For a "No" response in question 44.2, please answer the question "Is the [selected grain product] whole grain rich? (i.e. Foods that are considered "whole grain-rich" are a blend of whole-grain meal and/or flour and enriched meal and/or flour of which at least 50 percent is whole grain. The remaining 50 percent or less of grains, if any, must be enriched.)" with the answers "Yes", "No", or "I don't know".
    - 44.5 For a "Yes" response in question 44.4, please answer the question "How do you know the [selected grain product] is whole grain rich? (Select all that apply.) with the answers "By reading the ingredient list/label", "Food Program Sponsor", "Menu Planning Service", "On-site Kitchen/Food Manager/Cook", "I am not sure. I just think it is 100% whole wheat/grain.", and/or "The product is darker in color." Then move on to the next grain product in the list.

44. Does your site provide… (check all that apply)	44.1. For each grain provided, how often is it provided?	44.2. Is the product 100% whole wheat or whole grain?	44.3 How do you know that the product is 100% whole wheat or whole grain? (Select all that apply.)	44.4. If the product is not 100% whole wheat or whole wheat, is the product **whole grain rich?**	44.5 How do you know that the product is whole grain rich? (Select all that apply.)
☐ a. Whole wheat bread	☐ Twice a day or more<br>☐ Once a day<br>☐ 2-3 times/ week<br>☐ Once a week<br>☐ Less than 1x/week<br>☐ I don't know	☐ Yes **(GO TO QUESTION 44.3)**<br>☐ No (**GO TO QUESTION 44.4)**<br>☐ I don't know	☐ By reading the ingredient list/label<br>☐ Food Program Sponsor<br>☐ Menu Planning Service<br>☐ On-site Kitchen/Food Manager/Cook<br>☐ I am not sure. I just think it is 100% whole wheat/grain.<br>☐ The bread is brown in color.	☐ Yes **(GO TO QUESTION 44.5)**<br>☐ No<br>☐ I don't know	☐ By reading the ingredient list/label<br>☐ Food Program Sponsor<br>☐ Menu Planning Service<br>☐ On-site Kitchen/Food Manager/Cook<br>☐ I am not sure. I just think it is 100% whole wheat/grain.<br>☐ The bread is brown in color.
☐ b. Whole wheat pasta	☐ Twice a day or more<br>☐ Once a day<br>☐ 2-3 times/ week<br>☐ Once a week<br>☐ Less than 1x/week<br>☐ I don't know	☐ Yes **(GO TO QUESTION 44.3)**<br>☐ No (**GO TO QUESTION 44.4)**<br>☐ I don't know	☐ By reading the ingredient list/label<br>☐ Food Program Sponsor<br>☐ Menu Planning Service<br>☐ On-site Kitchen/Food Manager/Cook<br>☐ I am not sure. I just think it is 100% whole wheat/grain.<br>☐ The product is darker in color.	☐ Yes **(GO TO QUESTION 44.5)**<br>☐ No<br>☐ I don't know	☐ By reading the ingredient list/label<br>☐ Food Program Sponsor<br>☐ Menu Planning Service<br>☐ On-site Kitchen/Food Manager/Cook<br>☐ I am not sure. I just think it is 100% whole wheat/grain.<br>☐ The product is darker in color.
☐ c. Whole wheat tortilla	☐ Twice a day or more<br>☐ Once a day<br>☐ 2-3 times/ week<br>☐ Once a week<br>☐ Less than 1x/week<br>☐ I don't know	☐ Yes **(GO TO QUESTION 44.3)**<br>☐ No (**GO TO QUESTION 44.4)**<br>☐ I don't know	☐ By reading the ingredient list/label<br>☐ Food Program Sponsor<br>☐ Menu Planning Service<br>☐ On-site Kitchen/Food Manager/Cook<br>☐ I am not sure. I just think it is 100% whole wheat/grain.<br>☐ The product is darker in color.	☐ Yes **(GO TO QUESTION 44.5)**<br>☐ No<br>☐ I don't know	☐ By reading the ingredient list/label<br>☐ Food Program Sponsor<br>☐ Menu Planning Service<br>☐ On-site Kitchen/Food Manager/Cook<br>☐ I am not sure. I just think it is 100% whole wheat/grain.<br>☐ The product is darker in color.

44. Does your site provide… (check all that apply)	For each grain provided, how often is it provided?					
	Twice a day or more	Once a day	2-3 times/ week	Once a week	Less than 1x/week	I don't know
☐ d. Brown Rice	☐	☐	☐	☐	☐	☐
☐ e. White Bread	☐	☐	☐	☐	☐	☐
☐ f. White Rice	☐	☐	☐	☐	☐	☐
☐ g. Corn Tortilla	☐	☐	☐	☐	☐	☐
☐ H. None of the above.						

PLEASE USE THE TABLE TO ANSWER QUESTION 45

45. Which meat/protein-based products does your site provide children **ages 2-5 years**? (Select all that apply.)
    - For every meat/protein-based product selected in question 45, answer the question "How often does your site provide [selected meat/protein-based product] to children ages 2-5 years?" with the answers of "daily," "2-3 times per week," "once a week," "less than once a week (occasionally)," "rarely (special occasions only)," "I don't know".

	45. Does your site provide…(select all that apply)	For each meat/protein-based product provided, how often is it provided?					
		Daily	2-3 times per week	Once a week	Less than once a week (Occasionally)	Rarely (Special occasions only)	I don't know
☐	a. Packaged lunch meat (e.g. ham, turkey, or bologna)	☐	☐	☐	☐	☐	☐
☐	b. Beef or Pork Hot Dogs	☐	☐	☐	☐	☐	☐
☐	c. Turkey Hot Dogs	☐	☐	☐	☐	☐	☐
☐	d. Baked or Broiled Chicken, Turkey, or Fish	☐	☐	☐	☐	☐	☐
☐	e. Beef/Hamburger	☐	☐	☐	☐	☐	☐
☐	f. Breaded Chicken Nuggets/Strips	☐	☐	☐	☐	☐	☐
☐	g. Peanut Butter/Other Nut Butters or Spreads	☐	☐	☐	☐	☐	☐
☐	h. Other (Fill in blank):						

46. Please provide a copy of your site's monthly menu when you return the survey by mail.

Thank you for taking the time to complete the survey!

Please mail page 1-10 of the survey back in the provided envelope. Once we receive your completed survey, we will email you a $20 Amazon Electronic Gift Card, if you provide your email address (please do not provide a group email box) below. If you do not provide your email below, we will not send a gift card. Please provide a phone number to confirm receipt of the gift card. You should receive your electronic gift card within two weeks of us receiving your completed survey.

Email: ____ Phone Number: ______________________________________

For any questions about the survey that you just completed, please contact Dr. Jamie Chriqui at the University of Illinois at Chicago at JamieChriqui@uic.edu or 312-355-1353.
